# From Microbes to Metabolites: Advances in Gut Microbiome Research in Type 1 Diabetes

**DOI:** 10.3390/metabo15020138

**Published:** 2025-02-19

**Authors:** Lente Blok, Nordin Hanssen, Max Nieuwdorp, Elena Rampanelli

**Affiliations:** 1 Department of Internal and Vascular Medicine, Amsterdam University Medical Center, Location AMC, 1105 AZ Amsterdam, The Netherlands; n.m.j.hanssen@amsterdamumc.nl (N.H.); m.nieuwdorp@amsterdamumc.nl (M.N.); 2 Amsterdam Institute for Infection and Immunity (AII), Amsterdam, The Netherlands

**Keywords:** type 1 diabetes, gut microbiome, metabolites, short-chain fatty acids, beta cell function

## Abstract

**Background:** Type 1 diabetes (T1D) is a severe chronic T-cell mediated autoimmune disease that attacks the insulin-producing beta cells of the pancreas. The multifactorial nature of T1D involves both genetic and environmental components, with recent research focusing on the gut microbiome as a crucial environmental factor in T1D pathogenesis. The gut microbiome and its metabolites play an important role in modulating immunity and autoimmunity. In recent years, studies have revealed significant alterations in the taxonomic and functional composition of the gut microbiome associated with the development of islet autoimmunity and T1D. These changes include reduced production of short-chain fatty acids, altered bile acid and tryptophan metabolism, and increased intestinal permeability with consequent perturbations of host (auto)immune responses. **Methods/Results:** In this review, we summarize and discuss recent observational, mechanistic and etiological studies investigating the gut microbiome in T1D and elucidating the intricate role of gut microbes in T1D pathogenesis. Moreover, we highlight the recent advances in intervention studies targeting the microbiota for the prevention or treatment of human T1D. **Conclusions:** A deeper understanding of the evolution of the gut microbiome before and after T1D onset and of the microbial signals conditioning host immunity may provide us with essential insights for exploiting the microbiome as a prognostic and therapeutic tool.

## 1. Introduction

Type 1 diabetes (T1D) is a severe chronic autoimmune disease, affecting approximately 12.7 million people worldwide [1]. T1D is characterized by the destruction of the pancreatic beta cells, resulting in inadequate insulin production [2]. There is currently no curative treatment for T1D and exogenous insulin replacement is the only available treatment option for people living with T1D [3,4]. Despite significant advances in glucose monitoring technologies and insulin delivery systems, achieving glycemic targets remains challenging, with a staggering 73% of people with diabetes failing to achieve established glycemic targets [5,6,7,8]. T1D results in severe macrovascular and microvascular complications, manifesting as retinopathy, neuropathy and nephropathy [9]. These complications have a significant impact on life expectancy, with a global reduction of 24 years when comparing individuals with and without T1D, varying between 11 and 46 years in high- and low-income countries, respectively [1]. In addition, the incidence of T1D continues to rise at an alarming rate of 3–5% per year, resulting in a significant social and economic burden [10]. Epidemiological projections of T1D incidence suggest that prevalent cases will increase by 60–107% by 2040. The largest relative increases in T1D prevalence are projected to occur in low- and lower-middle-income countries, highlighting the growing inequity in the global distribution of the T1D burden [1]. These projections highlight the growing public health challenge posed by T1D and underscore the urgent need for a better understanding of the pathogenesis of T1D, as well as the development of improved treatment modalities and, ideally, effective prevention strategies. Accumulating evidence points to the gut microbiome as a crucial area of study for both understanding T1D pathogenesis and developing novel therapeutic approaches, that either target or exploit specific microbiota functions.

In the pathophysiology of T1D, T cell tolerance to pancreatic beta cell-derived autoantigens is known to be impaired [11]. Evidence from murine and human studies points to the pivotal role of autoreactive T cells in driving beta cell destruction and progression of T1D. In particular, autoreactive cytotoxic CD8+ T cells have been shown to infiltrate the pancreas and kill insulin-producing beta cells [12,13]. In addition to CD8+ T cells, CD4+ T cells are major players in the onset and progression of T1D, as they can produce pro-inflammatory cytokines in response to self-antigens, promote the activation of effector CD8+ T cells and macrophages, and provide help for B cells clonal expansion and antibody production against autoantigens [13]. Consequently, the pathogenesis of T1D is characterized by the development of cell-mediated and humoral autoreactive responses with the production of autoantibodies directed against beta cell-derived autoantigens, including insulin, glutamic acid decarboxylase (GAD), insulinoma-associated autoantigen-2 (IA-2) and zinc transporter-8 (ZnT8). Islet autoantibodies, although considered innocent bystanders, are diagnostic markers of T1D and are detectable in the circulation prior to the clinical manifestation of T1D [14]. The number of autoantibodies combined with the genetic risk score is the best predictor of the likelihood of developing T1D [15,16]. Although autoantibodies appear to be non-pathogenic, B cells together with dendritic cells serve as antigen-presenting cells that contribute to the initiation of the autoimmune attack against beta cells. While research has primarily focused on adaptive immunity, emerging evidence suggests that innate immune cells such as macrophages, natural killer cells and neutrophils also play a crucial role in the early stages and progression of T1D [17,18].

What triggers the autoimmune destruction of pancreatic B cells by autoreactive T cells is complex and its exact etiology is not fully understood [2]. Evidence from human studies indicates that T1D does not arise solely from a defective or incomplete central (thymic selection) tolerance, as healthy individuals also harbor autoreactive CD8+/CD4+ diabetogenic autoreactive T cells in the peripheral circulation. Nevertheless, homing of autoreactive T cells to the pancreas is a prominent feature of T1D individuals [19,20]. Genetic factors play an important role as familial linkage analysis and genome-wide association studies have discovered over 50 key genetic factors contributing to T1D susceptibility [21,22,23]. Particularly polymorphisms in Human Leukocyte Antigen (HLA) class II genes have emerged as the primary genetic determinants, with HLA variations such as HLA-DR3-DQ2 and HLA-DR4-DQ8 accounting for approximately 40–50% of familial aggregation of T1D [24,25]. However, genetics alone cannot explain the alarming increase in T1D incidence [26,27]. In addition, less than 10% of individuals with high-risk genotypes actually progress to develop T1D [28], and twin studies have shown a discordance in T1D development with concordance rates below 50% in monozygotic twins [29,30]. These findings highlight the critical role of environmental factors in triggering, modulating or accelerating the onset of T1D [26,27]. For example, there is evidence of an increased T1D risk associated with certain viral infections such as human enteroviruses, coxsackie B virus and rotavirus infections, but also with dietary influences such as early exposure to cow’s milk or vitamin D deficiency [31,32,33]. Recent research on T1D has increasingly emphasized the gut microbiome as a crucial environmental factor in disease pathogenesis, with numerous studies highlighting its potential role in modulating T1D susceptibility and progression, opening up potential strategies for prevention and intervention [34,35,36,37,38]. This review aims to outline current knowledge and significant findings regarding the role of the gut microbiome and microbiota-derived metabolites in T1D pathogenesis and treatment.

## 2. The Gut Microbiota, as “Educators” of the Immune System

The gut microbiome consists of the totality of microorganisms (bacteria, fungi, archaea, protozoa and viruses) living in the gut along with their genetic material, encoded proteins and metabolites and the number of microorganisms is estimated to exceed 10^14^ [39,40]. Nonetheless, most studies on gut microbiota largely investigate only the bacterial strains inhabiting the gastrointestinal tract, which harbors approximately 500–1000 bacterial species with the largest commensal communities residing in the colon [41]. Five bacterial phyla dominate based on 16S ribosomal RNA (16S rRNA) gene sequencing data: Firmicutes, Bacteroidetes, Proteobacteria, Actinobacteria and Verrucomicrobia [42]. This complex ecosystem residing in the gastrointestinal tract plays a crucial role in host’ homeostasis and disease, performing essential metabolic functions such as polysaccharide fermentation and vitamin biosynthesis [43]. The composition and diversity of microorganisms is unique between individuals and can be influenced by several factors, including geographic location, age, medication use, infections and diet [44,45,46]. Interestingly, HLA genotype also has a significant influence on the composition and diversity of the gut microbiome [47]. Despite the fact that the microbiota composition is unique to an individual and exhibits intra-individual fluctuations due to changes in diet, exercise, season, etc., a general definition of a “healthy” microbiome includes high taxonomic diversity, high microbial gene richness and a stable core microbiota [48]. Conversely, disease states are often associated with alterations in the taxonomic and functional composition of the microbiome, leading to an imbalance in the microbiota-derived beneficial or deleterious signals to the host, which may contribute to various disease conditions [49]. This perturbation may be due to depletion of beneficial bacterial populations, overgrowth of potentially pathogenic bacteria or the loss of overall bacterial diversity [50].

Exposure to the microbiota begins in utero and expands rapidly after birth, with mode of delivery and subsequent environmental exposures strongly influencing the composition of the microbiome [51]. Among various functions of the gut microbiome, commensal gut bacteria are crucial in the development of the host’s immune system and homeostasis [52]. In particular, early-life microbial colonization plays an important role in the development and training of immune cells [51]. Studies in germ-free (GF) mice have been critical in uncovering the fact that commensal microbes influence the development of the immune system and shape its function in adult life. Indeed, in the absence of microbial colonization, GF mice show deficits in the development of lymphoid organs including the gut-associated lymphoid tissue (GALT), such as smaller mesenteric lymph nodes and Peyer’s patches, and an imbalance in pro- and anti-inflammatory pathways mediated by IL-17-producing T helper cells (Th17) and Foxp3+ regulatory T cells (Tregs), respectively, reduced numbers of IgA-producing B cells and intraepithelial CD8+ T cells [53,54]. Together with immune defects, epithelial defense is disrupted in GF mice, with an altered mucosal layer and production of antimicrobial peptides (AMPs) [54,55]. Importantly, microbiota reconstitution was able to rescue immune cell and mucosal deficiencies in GF mice [56,57,58]. Given that the resident gut microbes are crucial for early life immune development as well as for maintaining immune homeostasis in adult life, it is not surprising that perturbations in the structure and function of resident microbial communities ultimately affect immune cell activity and the course of (auto)immune diseases. Specifically in T1D, an environment consistent with reduced tolerance has been found in the intestinal mucosa, with an increase in pro-inflammatory cytokines and a decrease in Tregs [59]. Furthermore, diabetogenic autoreactive T cells have been found to be primed in the gut of a T1D mouse model, while pancreatic draining lymph nodes serve as site of amplification in the autoimmune process [60].

## 3. Gut Microbiota in T1D

The hypothesis that microbial exposure significantly influences the development of T1D has been thoroughly investigated and confirmed by numerous studies in mouse models, in particular the non-obese diabetic (NOD) mouse, and in longitudinal human studies, such as the TEDDY study, DIABIMMUNE, ABIS, DIPP and INNODIA cohorts as well as smaller human studies [35,36,61,62,63,64,65,66,67,68,69,70,71,72,73,74,75]. The natural course of T1D is characterized by significant alterations in gut microbiota structure and function, both before and after the onset of clinical symptoms. Table 1 summarizes the main perturbations affecting the taxonomic composition of the microbiota in children and adults with T1D, at different stages of disease: asymptomatic in at-risk individuals, seroconversion, diabetes onset and long-standing T1D. Longitudinal cohort studies, such as the TEDDY, DIPP, INNODIA and DIABIMMUNE studies, have identified distinct microbial signatures in children who later develop islet autoimmunity and progress to T1D in a population of children that are genetically predisposed to T1D, or as in the ABIS study, have followed a “non-HLA restricted” general population from birth [64]. Microbial alterations have been observed at various stages of the disease, including seroconversion to islet autoimmunity, the period between seroconversion and T1D progression, new-onset T1D, and long-standing T1D. The diversity of gut microbiota in T1D patients has been reported to be either lower [36,74] or comparable to healthy controls [62], with no clear consensus. Interestingly, in the INNODIA study, the rate of subsequent disease progression was associated with a lower diversity, as individuals with a rapid decline in C-peptide levels, reflecting a decline in endogenous insulin production, had the lowest gut microbiome diversity [66].

The Environmental Determinants of Diabetes in the Young (TEDDY) cohort study collected blood and fecal samples from 783 children at genetic risk for T1D, from 3 months to 5 years of age, including children who seroconverted (n = 267), children diagnosed with T1D (n = 101) and controls (n = 415) [61]. Children with T1D showed a significant decrease in genes related to the fermentation and biosynthesis of short-chain fatty acid (SCFA) metabolites compared to healthy controls.

Overall, consensus has emerged regarding the association between increased abundance of *Bacteroidetes* and the development of islet autoimmunity and T1D [64,65,67,70,72,74,76], and a reduction in SCFA-producing bacterial species [64,66,67,68,72]. Comparison of children with at least two diabetes-associated autoantibodies with autoantibody-negative children in the TRIGR and FINDIA pilot studies showed that SCFA-producing species including *Bifidobacterium adolescentis*, *Roseburia faecis* and *Faecalibacterium prausnitzii* were negatively correlated with the number of autoantibodies detected [67]. Consistent with this, the TEDDY study observed a lower prevalence of microbial genes associated with fermentation and SCFA biosynthesis in individuals with T1D compared to healthy individuals [61]. Other notable alterations include a strong association between *Parabacteroides* abundance and T1D onset in a pediatric cohort [62], decreased abundance of *Bifidobacterium* and *Lactobacillus* [61,67,71], and mucin-degrading species such as *Akkermansia* at onset [62,72]. Although there are conflicting reports on the abundance of *Ruminococcus*, it was found to be a strong determinant in differentiating future T1D diagnoses from control cases [64]. Furthermore, functional remodeling of the gut microbiota has been shown to accompany islet autoimmunity and progression to T1D, including decreased bile acid metabolism [35,63,69] and increased lipopolysaccharide (LPS) biosynthesis [77]. In addition, deficiencies in proteins involved in intestinal epithelial barrier function, microvilli adhesion and exocrine pancreatic function are found in both T1D patients and those at high genetic risk for T1D, suggesting that the intestinal barrier in T1D patients is impaired prior to the onset of islet autoimmunity [35,69]. Furthermore, specific gut microbiome compositions have been associated with glycemic control, glycated hemoglobin (HbA1c) levels and disease duration, as well as micro- and macrovascular complications, explaining a significant proportion of variation in the gut microbiome [66,68,70,71].

## 4. Potential Mechanisms by Which Gut Microbes Shape the Course of T1D

**Figure 1:**
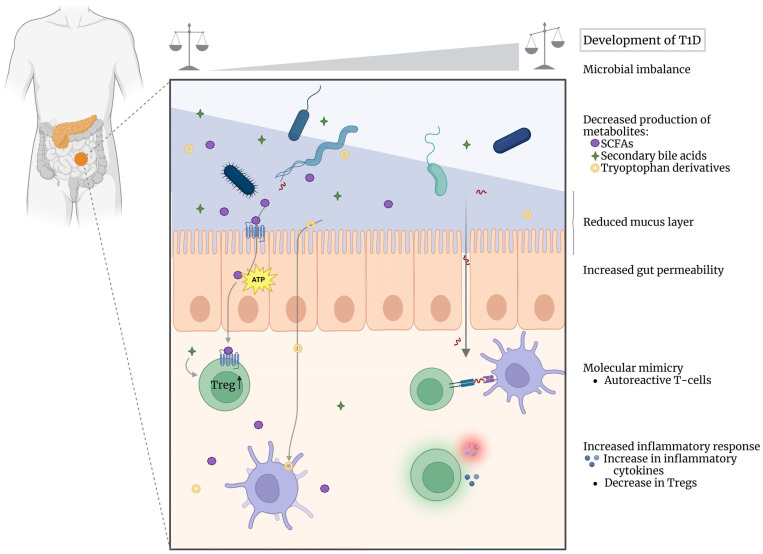
**Schematic representation of the pathological mechanisms by which alterations in the gut microbiome can lead to T1D onset or exacerbate systemic inflammation and autoimmune responses.** We visualized a spectrum from lower to higher risk of developing T1D, from left to right. Under homeostatic conditions (left side of the spectrum), a ’healthier’ and diverse microbiome produces a wide range of metabolites, such as SCFAs, secondary bile acids and tryptophan derivatives, fostering the intestinal barrier and leading to a more tolerogenic environment with stimulation of Treg differentiation. In contrast, a more imbalanced microbiome (right side of the spectrum) can perturb the production of microbial metabolites, and increase gut permeability with a reduced protective mucus layer. This leads to an enhanced inflammatory response with increased secretion of pro-inflammatory cytokines and decreased levels of intestinal Tregs, indicating a less tolerogenic environment. In addition, gut microbes that express peptides with similarities to pancreatic beta cell epitopes can trigger the activation of autoreactive T cells (via molecular mimicry). Altogether, these effects may lead to or exacerbate the autoimmune responses that drive beta cell destruction and the development of T1D. Figure created using BioRender (https://www.biorender.com/, accessed on 12 February 2025).

The gut microbiome is hypothesized to influence the pathophysiology of T1D through multiple mechanisms, and a comprehensive understanding of these mechanisms is crucial for the development of targeted therapeutic interventions. These proposed mechanisms encompass a range of microbial-host interactions, including the production of bioactive metabolites, modulation of intestinal barrier function, and potential molecular mimicry between microbial and host antigens (Figure 1).

### 4.1. Metabolites

The gut microbiome possesses a diverse array of enzymatic capabilities, enabling the fermentation of a wide range of compounds that are either incompletely indigested or indigestible by human enzymes such as dietary fibers. As a result, gut microbiota produce metabolites with diverse bioactivities that can interact directly with host intestinal cells or be absorbed into the circulation and influence systemic immune responses [78]. Metabolites derived from the gut microbiome are crucial mediators of host-microbial interactions [79]. Previous studies have shown that dysregulated microbial metabolites were associated with the manifestation of type 1 diabetes [80,81]. Furthermore, when comparing gut microbiome data from children with T1D and healthy controls across geographically diverse clinical centers, it is taxonomically more diffuse, but functionally more coherent [61], suggesting that the focus should be more on the functionality of the gut microbiota rather than its taxonomic composition. Key metabolites associated with T1D and autoimmunity include SCFAs, tryptophan metabolites and bile acids.

#### 4.1.1. Short-Chain Fatty Acids

Microbiota-derived SCFAs are metabolites that are produced from non-digestible dietary fibers, and consist mainly of acetate, propionate and butyrate [82]. The production of SCFAs depends on the microbiota composition, fiber content of the host diet, intestinal transit time and the metabolic flux of SCFAs between host and microbiota [83]. SCFAs are the major energy source for colonic epithelial cells, as colonocytes derive 60–70% of their energy supply from SCFA oxidation [84]. SCFAs interact with metabolite-sensing G protein-coupled receptors (GPCRs) expressed in the intestinal epithelium and immune cells and inhibit histone deacetylases (HDACs) [85]. SCFAs are critical for maintaining gut homeostasis through their metabolic and anti-inflammatory functions, improving gut epithelial barrier function and promoting immune tolerance. Epithelial barrier integrity is strengthened by stimulating intestinal goblet cells to increase mucus production and by upregulating tight junction proteins [86,87]. SCFAs can modulate the immune system by influencing differentiation of Treg and Th17 cells and by decreasing the proliferation and cytokine production of T helper 1 (Th1), Th17 and T helper 22 (Th22) cells via proliferation-activated receptor γ (PPARγ), thereby influencing tolerance to gut microbiota [88]. Butyrate and propionate can induce extrathymic generation and differentiation of Treg cells through HDAC inhibition [89]. In addition, SCFA butyrate supplementation inhibits the responsiveness to LPS-induced production of pro-inflammatory cytokines interleukin-6 (IL-6) and interleukin-12 (IL-12) secretion in intestinal macrophages [90]. Gut-derived SCFAs can reach the pancreas, where they control the production of cathelicidin-related antimicrobial peptides (CRAMP), which in turn induce Treg generation in the pancreatic islets, resulting in an immunosuppressive environment that limits autoimmunity [91]. In vitro, SCFAs can increase insulin levels and preserve C-peptide levels in human beta-like cells and in a NOD/SCID (severe combined immunodeficiency disease) mouse model, SCFAs can improve the differentiation of human embryonic stem cells into beta-like cells [92].

Blood and fecal acetate and butyrate levels were found to be inversely correlated with insulitis and disease progression in NOD mice [93,94]. Early life administration of these SCFAs via diets containing high-amylose maize starch (HAMS) that have been acetylated (HAMSA) or butyrylated (HAMSB) reduced insulitis and protected NOD mice from developing T1D [93]. Administration of butyrate increased Tregs in the colon, mesenteric lymph nodes and Peyer’s patches and led to migration of Tregs from the GALT to the pancreatic lymph nodes and pancreas, delaying the onset of diabetes [95]. Treatment with acetate reduced gut bacteria-induced IgA responses accompanied with a reduced severity of insulitis in NOD mice [96]. In addition, butyrate supplementation was found to reduce serum IL-1β, improve islet morphology, reduce inflammatory cell infiltration, and restore Th1/Th2/Th17 levels compared to control [97]. Even maternal supplemented butyrate was able to protect NOD mouse offspring from developing T1D [98]. Dietary intervention with the indigestible soluble fiber inulin was used to promote production of SCFAs and SCFA-producing bacteria in a T1D mouse model, which also resulted in enhanced mucus production, reduced pancreatic inflammation and enhanced Treg function [94]. Additionally, a combination of the SCFAs butyrate and acetate was shown to have an enhancing effect compared to monotherapy, suggesting different cellular or molecular pathways of these SCFAs [93]. Mice fed HAMSAB (combination of acetylated and butyrylated HAMS) showed pancreatic islets with a more immune-tolerogenic phenotype with preservation of pancreatic endocrine cell identity [92]. Thus, SCFAs influence tolerance of the immune system by expanding the pool of Tregs and can harness T1D pathogenesis in murine models.

#### 4.1.2. Tryptophan Metabolites

Tryptophan catabolites produced by the host or by gut microbiota play a crucial role in maintaining intestinal and systemic immune homeostasis [99,100]. Degradation of the essential amino acid tryptophan occurs via three main pathways: the kynurenine pathway, the serotonin pathway and the indole pathway. While the first two are major degradation pathways that occur in mammalian cells, the indole pathway is typical of commensal bacteria and generates a wide array of bacterial metabolites (indole derivatives) through the action of the bacterial enzymes TMO (tryptophan 2-monooxygenase), TrD (tryptophan decarboxylase), ArAT (aromatic amino acid aminotransferase), and TNA (tryptophanase) and homologs of eukaryotic indoleamine 2,3-dioxygenase 1 (IDO) [101]. Tryptophan metabolism has been identified as a key factor in the regulation of immune balance, and its dysregulation contributes to the development of T1D in both NOD mice and human patients [102,103,104]. In particular, the “kynurenine pathway” of tryptophan metabolism is now recognized as an important immune checkpoint, generating downstream metabolites that suppress effector T cell function and favor differentiation of Tregs [105,106]. In light of the important immunoregulatory role of tryptophan derivatives, the early stages of islet dysfunction in T1D, preceding the autoimmune attack, are characterized by loss of expression of IDO1 in beta cells, which catalyzes the degradation of tryptophan to kynurenine, thereby promoting immunotolerance [101,104]. Gut microbiota can indirectly influence the kynurenine pathway of tryptophan metabolism by regulating host IDO1 activity through other microbiota-derived metabolites, such as SCFAs [106]. It was shown that butyrate negatively regulates IDO1 expression in intestinal epithelial cells by interfering with STAT1 (signal transducer and activator of transcription 1) signaling and histone deacetylation of the IDO1 promoter, suggesting a potential role for gut microbiota-derived metabolites in regulating the kynurenine pathway [107]. Consistently, the absence of gut microbiota in GF mice was shown to reduce host IDO activity as assessed by a decreased plasma kynurenine/tryptophan ratio and colonization of GF mice could restore the plasma kynurenine/tryptophan ratio to control values [108].

At the same time, gut bacteria can influence tryptophan metabolism via the “indole pathway” using the enzyme tryptophanase to produce derivatives such as indole-3-aldehyde (IAld), indole-3-acid-acetate (IAA), indole-3-propionic acid (IPA), indole-3-acetaldehyde (IAAld), and indoleacrylic acid (IA) [106]. These indole derivatives act as ligands for the aryl hydrocarbon receptor (AhR). AhR is expressed by intestinal epithelial cells and drives epithelial cell renewal and turnover, but is also expressed by intestinal immune cells, including antigen presenting cells (APCs), innate lymphoid cells (ILCs) and Th17/Th22 cells [109]. Activation of the tryptophan-AhR axis in the gut induces the expression of cytokines like IL-22 and IL-17, which promote intestinal homeostasis [100]. AhR activation leads to the induction of IL-22, which stimulates mucosal defense by promoting production of AMPs and enhancing goblet cell proliferation for mucin secretion [110]. Additionally, bacterial indoles can improve epithelial barrier function through the pregnane X receptor (PXR), reducing production of the pro-inflammatory cytokine tumor necrosis factor-α (TNF-α) and improving epithelial goblet cell function [111]. Changes in diet or microbiota structure can greatly influence AhR activity. Indeed, a reduction in gastrointestinal tryptophan availability or changes in the population of indole-producing bacteria can result in decreased indole production, leading to reduced stimulation of AhR and PXR. Consequently, AhR-mediated regulation of immune function is impaired and intestinal barrier integrity is compromised, ultimately contributing to the development of chronic inflammation [106]. In NOD mice, reduced AhR activity was observed, but when activated, it led to an expansion of Foxp3+ Treg cells and reduced pancreatic insulitis [112,113]. In T1D patients, tryptophan catabolites, such as kynurenine, are significantly decreased compared to healthy controls and their abundance can be rescued by probiotic administration, such as *Lactobacillus rhamnosus* GG [100,114]. Also, when comparing the metabolome of anti-GAD65 antibody positive and anti-GAD65 antibody negative T1D patients (52 positive versus 49 negative), metabolites related to tryptophan metabolism were found to be significantly different, including 3-hydroxyanthranilic acid, L-phenylalanine, and L-kynurenine, as they were found to be reduced in patients with anti-GAD65 autoantibodies compared to patients with negative anti-GAD65 antibodies. In addition, the anti-GAD65 antibody positive group also showed an enrichment of the bacterial genera *Alistipes* and *Ruminococcus* [115]. Furthermore, the microbiota-derived tryptophan metabolite IAA was found to be significantly different in children from the Finnish Type 1 Diabetes Prediction and Prevention (DIPP) study who progressed rapidly to T1D after seroconversion compared to those who progressed slowly [103].

#### 4.1.3. Secondary Bile Acids

Bile acids, traditionally recognized for their crucial role in facilitating the absorption of lipids and fat-soluble vitamins, have emerged as important regulators of metabolic processes and innate immunity. Interestingly, gut microbes interact with bile acid metabolism by converting primary bile acids into secondary bile acids in the gut [116]. These secondary bile acids can act as metabolic regulators because they are ligands for receptors in various peripheral tissues, including the farnesoid X receptor (FXR) and the Takeda G protein-coupled membrane receptor (TGR5). Both receptors are important in the regulation of glucose homeostasis and insulin sensitivity. TGR5 can inhibit the release of cytokines from macrophages after exposure to LPS [117]. In addition, secondary bile acids can act as ligands of retinoid-related orphan receptor-γt (RORγt), a key transcription factor of Th17 cells, thereby influencing Th17 cell development and function [88]. Bile acid metabolites modulate the differentiation and function of Tregs through the production of mitochondrial reactive oxygen species (ROS), leading to increased expression of Foxp3 [118]. In a mouse model of early-stage T1D, administration of tauroursodeoxycholic acid (TUDCA), the conjugated form of the secondary bile acid ursodeoxycholic acid (UDCA), showed a 43% reduction in blood glucose and an increase in beta cell mass, which may be a result of reduced immune cell infiltration into the pancreatic islets, as TUDCA has also been shown to prevent insulitis in pre-diabetic NOD mice [119,120]. In humans, a decrease in bile acid metabolism was found in children with new-onset T1D compared to healthy controls [35]. Dysregulation of secondary bile acid metabolism has also been shown to precede islet autoimmunity in children who go on to develop T1D [63]. This was associated with decreased abundance of *Clostridium* species and *Eggerthella lenta* and increased abundance of *Runinococcus* strains at 18 and 24 months of age in children with two or more islet autoantibodies [63].

Overall, these studies suggest that altered bile acid metabolism in early life may disrupt immune homeostasis and contribute to the pathogenesis of T1D.

### 4.2. The “Leaky” Gut

The function of the intestinal barrier has been shown to be a crucial factor in the interplay between the gut microbiome and the host, acting as a gatekeeper between the human body and the luminal contents with commensal bacteria, pathogens, bacterial products and food antigens [121]. The gut barrier is composed of a mucus layer, a protective layer containing immunoregulatory molecules such as antimicrobial peptides, and an intestinal epithelial barrier held together by a junctional system of transmembrane and scaffolding proteins [121]. Aberrant intestinal barrier integrity has been observed in both preclinical and clinical studies of T1D in both the prediabetic and diabetic stages [35,59,69,121]. In preclinical studies, interventions that accelerate the onset of diabetes typically also increase intestinal permeability [59]. In the case of increased intestinal permeability (so-called “leaky gut”), the intercellular spaces between intestinal epithelial cells become wider due to increased bioavailability of zonulin, a physiological modulator of intercellular tight junctions, and downregulation of the expression of genes encoding tight junction proteins, both of which are found prior to the diagnosis of T1D [122,123]. Low-grade intestinal inflammation is also observed, with reduced mucosal production of IL-17A, IL-22 and IL-23A, which are important for host mucosal defense and barrier function [124]. Loss of barrier integrity allows bacterial products and diabetogenic antigens to engage intestinal mucosal immune cells and eventually enter the systemic circulation and the lymphatic system [121]. In a mouse model, it has been elegantly shown that disruption of intestinal barrier integrity by low-dose dextran sulfate sodium (DSS) in NOD mice carrying a transgenic T cell receptor specific for beta cell autoantigen leads to activation of islet-reactive T cells in the mesenteric lymph node, which subsequently migrate to the pancreatic lymph nodes and islets to mediate beta cell damage and T1D [121]. Importantly, this activation of islet autoimmunity requires the presence of gut microbiota, as depletion of commensal microbiota by antibiotic treatment abolished this process. Another study in streptozotocin-treated mice showed that an altered gut microbiome and loss of intestinal barrier function, together with a translocation of bacteria to the pancreatic lymph node, led to disease pathogenesis by inducing T helper Th1 and Th17 cells in the pancreatic lymph nodes, a process that was abolished by broad-spectrum antibiotics [125].

Human subjects with islet autoimmunity had a significant increase in intestinal permeability as measured by the lactulose-mannitol test compared to healthy controls [126]. This was observed in preclinical, new-onset and long-standing T1D patients, suggesting a subclinical enteropathy detectable at the stage of seroconversion prior to clinical diagnosis. Furthermore, alterations in gut microbiome composition are associated with loss of gut barrier function, creating a vicious cycle that exacerbates gut permeability and inflammation [123]. Microbial products such as SCFAs also play an important role in maintaining an intact intestinal barrier by promoting mucus secretion from goblet cells, regulating inflammatory and immune responses, and influencing the expression of tight junction proteins [86,127]. Indoles also contribute to the maintenance of the intestinal barrier by increasing mucus production and upregulating the expression of cell junction-related molecules through PXR activation [127]. Interestingly, administration of an anti-inflammatory diet enriched in omega-3 polyunsaturated fatty acids and inulin to NOD mice resulted in increased mucus layer thickness and upregulation of structural and immunoregulatory mucins and tight junction proteins, restoring gut barrier integrity, together with an increase in mucus-modulating bacteria *Akkermanisa muciniphila* and *Akkermansia glycaniphila* [128]. This not only led to a reduction in intestinal inflammation and expansion of Tregs in the gut, pancreatic lymph nodes and intra-islet lymphocytes, but also prevented the onset of T1D. These findings highlight the complex interplay between gut barrier function, microbiome composition, and T1D pathogenesis, and suggest potential therapeutic avenues through dietary interventions and microbiome modulation.

### 4.3. Molecular Mimicry

Another proposed mechanism by which gut microbes may trigger or exacerbate autoimmunity in T1D is the process of molecular mimicry, in which a foreign antigen shares sequence or structural similarities with self-antigens [129]. Gut microbes can express peptides that share similarities with epitopes of host beta cell autoantigens (mimotopes) [129,130]. This molecular similarity can lead to the activation of cross-reactive T cells that recognize both bacterial and beta cell-derived antigens, potentially triggering or exacerbating the autoimmune attack on pancreatic islets. For example, the hprt4-18 peptide from the human gut commensal *Parabacteroides distasonis* not only shares >50% homology with the insulin B-chain 9-23 sequence (insB:9-23), but also activates HLA-DQ8 CD4+ T cells from T1D patients in vitro [131]. Furthermore, colonization of female NOD mice with *P. distasonis* accelerated the development of T1D in a T cell-mediated manner, increasing macrophages, dendritic cells, and destructive CD8+ T cells, while decreasing FoxP3+ Tregs. Interestingly, these immune responses were not the result of general gut barrier dysfunction, as LPS levels were not significantly altered. Furthermore, re-analysis of gut microbiome data from children in the longitudinal DIABIMMUNE study showed that both autoantibody formation and disease progression were increased in children whose microbiomes harbored the hprt4-18 peptide of *P. distasonis* compared to children who did not harbor sequences of this peptide [131,132]. In another study, Tai and colleagues discovered a microbial mimic peptide derived from a magnesium transporter (Mgt) protein of intestinal *Leptotrichia goodfellowii*, a member of the phylum *Fusobacteria*, which was found to express a mimitope peptide within the Mgt protein that mimics the IGRP-derived self-epitope. In NY8.3 NOD mice expressing the islet-specific glucose-6-phosphatase catalytic subunit–related protein (IGRP)–reactive CD8+ T cell receptor, both the microbial mimitope and the *Fusobacteria* itself activate IGRP_206–214_-specific NY8.3 CD8+ T cells and drive T1D development [133]. The study of Nanjundappa et al. also revealed the existence of a low-avidity mimitope derived from integrase expressed by several species of the gut microbial genus *Bacteroides* that mimics the beta cell autoantigen IGRP_206–214_. Using integrase-competent, integrase-deficient, or integrase-transgenic (overexpressing) *Bacteroides* to colonize GF mice, the authors showed that the integrase-derived epitope promotes the recruitment of diabetogenic CD8+ T cells to the gut and suppresses DSS-induced colitis by killing autoantigen-loaded dendritic cells. However, the ability of this mimitope to either exacerbate insulitis or direct T cells to the intestinal mucosa, and hence be protective in T1D were not further investigated [134].

More recently, using a novel comprehensive immunoinformatics analysis of T1D-specific T cell epitopes, a number of intestinal pathogens and commensals have been identified as potent mimitopes across bacterial, viral and fungal proteomes [130]. This highlights an underestimated role of bacterial protein mimics in T1D pathogenesis and provides a promising starting point for microbiome-based therapeutics.

## 5. Microbiome-Based Human Interventions for T1D

**Figure 2:**
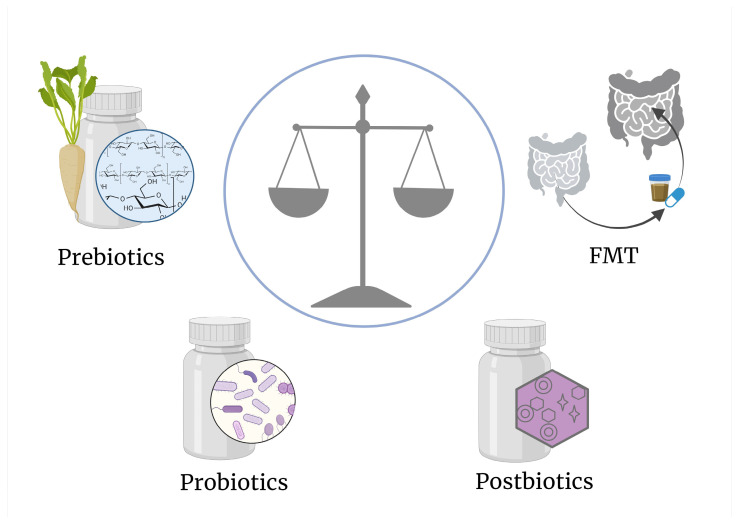
**Possible strategies to target the gut microbiota and shape the microbiota-host immunity crosstalk in T1D.** These include administration of substrates, which will be metabolized by specific microbes (prebiotics), of live beneficial bacteria conferring health benefits (probiotics), or of functional bioactive compounds derived by microbial fermentation (postbiotics). Another strategy, which encompasses all of the above, is whole fecal microbiota transplantations (FMT), administered as a filtered fecal suspension or via encapsulated lyophilized bacteria. Figure created using BioRender.

Several approaches can be used to modulate the gut microbiome and potentially influence the immune system and pathogenesis of T1D. These strategies encompass the rational use of (broad-spectrum) antibiotics, especially early in life; dietary interventions such as fiber-rich diets or specific dietary patterns; prebiotics that stimulate growth and function of beneficial bacteria; specific bacterial strains as probiotics or their metabolites (postbiotics); and fecal microbiota transplantation (FMT) (Figure 2). These interventions aim to reshape the gut microbiome, potentially mitigating immune dysregulation and influencing T1D progression through various mechanisms, including the production of SCFAs, improvement of gut barrier function, and modulation of host immune responses.

### 5.1. Prebiotic-Based Interventions

Prebiotics are “substrates that are selectively utilized by host microorganisms conferring a health benefit” [135]. Common prebiotics are dietary fibers such as fructo-oligosaccharides (FOS), galacto-oligosaccharides (GOS) and inulin [136]. The health benefits of prebiotics are specifically linked to their fermentation by gut microbiota, which produces SCFAs and other beneficial metabolites. In children with established T1D, prebiotic supplementation with oligofructose-enriched inulin for 12 weeks not only affected microbiome composition, but also resulted in significant preservation of C-peptide levels compared to placebo, along with a significant improvement in gut permeability as measured by lactulose/mannitol test, see Table 2 [137]. Notably, this intervention did not lead to changes in glycemic control or inflammatory markers. However, in the DIPP prospective birth cohort study of 5455 children with genetic susceptibility to T1D, non-specified dietary fiber intake was associated with the risk of developing islet autoimmunity (hazard ratio 1.41; 95% CI, 1.10–1.81) [138]. On the other hand, dietary fiber intake may improve glycemic control in patients with T1D, as in the GUTDM1 cohort study of 470 T1D patients a higher dietary fiber intake was independently associated with a longer period of time in euglycemic range, a marker of glycemic control [139]. Moreover, when comparing 24-week low-fiber and high-fiber diets, a reduction in mean daily blood glucose and HbA1c levels was observed [140].

### 5.2. Probiotic-Based Interventions

Probiotics are “live microorganisms that, when administered in adequate amounts, confer a health benefit on the host” [141]. Several *Lactobacillus* and *Bifidobacterium* species are generally considered to be safe microorganisms and are widely used, including in intervention studies in T1D patients. The effect of different single or multiple strain probiotics, with durations varying between two and six months in patients with new-onset or long-standing T1D was studied in several clinical trials, focusing on glycemic and immunological parameters as well as gut microbiome composition and possible underlying mechanistic pathways, see Table 3. The study of Wang et al. compared multi-strain probiotic supplementation for 6 months in a randomized, double-blind, placebo-controlled trial in children with T1D and observed significant changes in microbiome composition with significantly increased *B. animalis*, *L. slaivarius* and *Akkermansia* compared to the control group, as well as an improvement of glycemic parameters with reduced fasting blood glucose and HbA1c levels in the probiotic group, possibly mediated by immunomodulation, as several inflammatory cytokines were significantly decreased [142]. Furthermore, probiotic intervention studies in T1D patients have also investigated tryptophan metabolism in relation to immunomodulatory effects. Mondanelli et al. demonstrated that daily oral probiotic supplementation with *Lactobacillus rhamnosus* GG significantly affected circulating tryptophan levels and metabolite formation patterns in T1D patients while reducing inflammatory cytokines, confirming the importance of the role of microbiota-regulated tryptophan metabolites [100].

Four clinical trials studied the effect of probiotics on residual beta cell function by measuring (area under the curve) C-peptide levels, reflective of endogenous insulin production, in children with new-onset T1D [143,144,145,146]. *Lactobacillus rhamnosus* GG and *Bifidobacterium lactis* Bb12 for 6 months and a multi-strain probiotic product from VIVOMIXX^®^ for 3 months did not alter C-peptide levels compared to placebo [144,145]. Although the combination of *Lactobacillus rhamnosus* GG and *Bifidobacterium lactis* Bb12 showed no effect on glycemic control, the use of VIVOMIXX^®^ showed improved HbA1c levels and reduced total insulin requirements [145]. Interestingly, Lokesh et al. also compared VIVOMIXX^®^ (n = 27) with placebo (n = 23) in children with new-onset T1D, but over a period of 6 months. Here, a significant increase in C-peptide levels as well as a significant decrease in median HbA1c levels were found in the probiotics group while C-peptide levels declined as expected in the placebo group, indicating preservation or recovery of residual beta cell function along with better glycemic control in the probiotics group [143]. This may be due to immunomodulation, as there was a significant increase in the percentage of induced Treg cells, an increase in plasma IL-10 levels and a decrease in anti-IA2 antibodies, but the mechanism is not fully understood [143]. The discrepancy in results observed with the same probiotic product may be attributed to the extended duration of the intervention and is, to date, the first human study that to demonstrate an increase in residual beta cell function after probiotic supplementation [147].

A different approach was used by genetically engineering *Lactococcus lactis* AG019 ActoBiotics™, designed to deliver the self-antigen proinsulin and the human cytokine IL-10 to the gastrointestinal mucosa, with the aim of promoting immune tolerance [146]. Here, probiotic AG019 supplementation for 8 weeks was studied both as a monotherapy and in combination with the CD3 monoclonal antibody teplizumab for 12 days compared to placebo in adults and adolescents with recent onset of T1D. Both C-peptide levels and exogenous insulin use stabilized with probiotic monotherapy and combination therapy at 6 months and 12 months post-treatment, demonstrating a response to probiotics independent of teplizumab co-administration. In addition, a reduced frequency of preproinsulin-specific CD8+ T cells was found, suggesting that administration of the engineered bacterium modulated immune tolerance by reducing the production of specific Tregs, which could potentially reduce or eliminate pancreatic beta cell destruction [146].

Probiotics could also be used as a preventive therapeutic strategy. The TEDDY study group reported a significant association between early supplementation with probiotics and reduced risk of islet autoimmunity in children at high genetic risk for T1D who were followed from birth to 10 years of age [61,148]. It was found that exposure to probiotics in the first month of life, either from food supplements or infant formula, was associated with a 34% reduction in the risk of developing islet autoimmunity compared with later probiotic exposure or no probiotic supplementation (hazard ratio 0.66; 95% CI, 0.46–0.94). In addition, early probiotic exposure was associated with a 60% reduction in the risk of developing islet autoimmunity in children with the DR3/4 genotype, but not in other genotypes. Of note, most of the supplements contained *Lactobacillus* and *Bifidobacterium* species, but the species and amounts of microbes from probiotics are not defined and thus these interesting findings cannot be corroborated in controlled trials using mono- or combination therapies.

However, different findings emerged from a randomized, double-blind placebo controlled clinical trial designed to study the prevention of allergy in a cohort of 1223 children at high risk of allergy. Here, capsules of placebo or of a probiotic preparation containing *Lactobacillus* GG, *Lactobacillus rhamnosus* LC705, *Bifidobacterium breve* Bb99, and *Propionibacterium freudenreichii* ssp. *shermanii* JS were given to mothers from the 36th week of pregnancy and to infants from birth until 6 months of age. At follow-up visits, 25 children tested positive for autoantibodies at the age of 5 years and 14 children were T1D diagnosed at the age of 13 years, of whom 7 had received the active probiotics. Hence, within this small group of children, there was no significant impact of probiotic interventions in infancy on the development of T1D by 13 years of age or the cumulative incidence of beta cell autoimmunity by 5 years of age [149]. These findings suggest that early probiotic supplementation may play a role in modulating the immune system and potentially reducing the risk of T1D-associated autoimmunity specifically in genetically susceptible infants. Another study targeting early life microbiota is the SINT1A trial, a comprehensive clinical trial, initiated by the Global Platform for the Prevention of Autoimmune Disease, designed to investigate the potential of *Bifidobacterium infantis* supplementation in preventing the onset of T1D in infants at increased genetic risk (>10%) for T1D, as determined by genetic risk score or family history and HLA genotype. [150]. This randomized, placebo-controlled, double-blind study spans multiple centers and countries and focuses on primary prevention as interventions (placebo or *B. infantis* EVC001) starts between 7 days and 6 weeks of age and continues until 12 months of age. The primary outcome is to assess whether daily probiotic supplementation can reduce the development of multiple beta cell autoantibodies in children by the age of 6 years [150].

**Table 3:** Summary of main clinical studies using live probiotics as intervention for treatment or prevention of T1D.

Intervention	Population	Main Changes	Reference
*Lactobacillus salivarius* subsp. *salicinius* AP-32, *L. johnsonii* MH-68, and *Bifidobacterium animalis* subsp. *lactis* CP-9I supplementation vs. placebo for 6 months. Randomized, double-blind, placebo-controlled trial.	Children with T1D (probiotic n = 27; placebo n = 29) with mean T1D duration of 74.4 ± 53.6 months. Age 14.1 ± 5.1 years.	*B. animalis*, *L. slaivarius* and *Akkermansia* were significantly higher in the probiotic group, while *Akkermansia* and *Verrucomicrobia* were reduced in both groups. Fasting blood glucose levels and Hba1c were significantly reduced in the probiotic group. Inflammatory cytokines IL-8, TNF-alpha, IL-17, MIP-1β and RANTES were significantly reduced in the probiotics group, whereas TGF-beta1 was increased.	Wang et al., 2022 [142]
*Lactobacillus rhamnosus* GG and *Bifidobacterium lactis* Bb12 supplementation vs. placebo for 6 months. Randomized, double-blind, placebo-controlled trial.	Children with newly diagnosed T1D (probiotic n = 48; placebo n = 48). Age 12.3 ± 2.1 years.	No significant difference in (area under the curve) C-peptide levels. No significant differences in cytokine or zonulin levels.	Groele et al., 2021 [144]
*Bifidobacterium longum*, *Lactobacterium bulagricumi*, *Streptococcus thermophilus* supplementation vs. placebo for 12 weeks. Randomized, double-blinded, placebo-controlled.	Adults with T1D (probiotic n = 27, placebo n = 23).	Significant reduction in fasting blood glucose and postprandial glucose. No significant difference in HbA1c levels and CGM parameters.	Zhang et al., 2023 [151]
*L. paracasei*, *L. plantarum*, *L. acidophilus*, and *L. delbrueckii* subsp. *bulgaricus*, *B. longum B. infantis*, *B. breve* and *Streptococcus thermophilus* (VIVOMIXX^®^) supplementation vs. placebo for 3 months. Randomized, double-blind, placebo-controlled pilot study.	Children with new-onset T1D (probiotic n = 45, placebo n = 45). Age 7.9 ± 3.9 years.	Significant reduction in HbA1c levels. No significant changes in C-peptide, but significant reduction in insulin requirements in the probiotic group compared to placebo.	Kumar et al., 2021 [145]
*Bifidobacteriumlactis*, *Bifidobacteriumbifidum*, *Lactobacillus acidophilus* and *Lactobacillus ramenus* supplementation vs. placebo for 90 days. Randomized, single-blind, placebo-controlled trial.	Children with T1D with onset of disease within last 3 years (probiotic n = 26, placebo n = 26). Age 9.3 ± 2.9 years.	No significant effect on HbA1c levels. Significant decrease in fasting blood glucose level in probiotic group compared to control.	Shabani-Mirzaee et al., 2023 [152]
*L. paracasei*, *L. plantarum*, *L. acidophilus*, and *L. delbrueckii* subsp. *bulgaricus*, *B. longum B. infantis*, *B. breve* and *Streptococcus thermophilus* (VIVOMIXX^®^) supplementation vs. placebo for 6 months. Randomized, double-blinded, placebo-controlled trial.	Children with new-onset T1D (probiotic n = 27, placebo n = 23)	Significant decrease in median HbA1c levels and significant increase in median C-peptide levels in the probiotic group, as well as a significant increase in the percentage of induced Tregs. After probiotics, a significant increase in plasma IL-10 levels and a decrease in anti-IA2 were found in the probiotics group compared to control.	Lokesh et al., 2024 [143]
*Lactobacillus rhamnosus* GG *supplementation* vs. placebo for 3 months. Randomized, placebo-controlled study.	Children with T1D (probiotic n = 36, placebo n = 25). Age 3–18 years.	Significant increase in circulating tryptophan and decrease in pro-inflammatory cytokines (IFN-gamma, IL-17) in the probiotic group compared to placebo.	Mondanelli et al., 2020 [100]
*Lactococcus lactis* genetically modified to express human proinsulin and human IL-10 (AG019 ActoBiotics™) supplementation as monotherapy, in combination with teplizumab (CD3 monoclonal antibody) vs. placebo for 8 weeks. Randomized, open-label, placebo-controlled study	Adults and adolescents with recent onset T1D (monotherapy n = 8, placebo n = 8, combination n = 14, placebo n = 3). Age 12–42 years.	C-peptide and insulin use stabilized with probiotic monotherapy at 6 months and Hba1c at 12 months post treatment initiation. C-peptide, insulin use and Hba1c stabilized or improved with combination therapy up to 12 months. Significant reduction in the frequency of preproinsulin-specific CD8+ T cells after treatment with monotherapy or combination therapy.	Mathieu et al., 2023 [146]

### 5.3. Synbiotic-Based Interventions

Synbiotics, which combine prebiotics and probiotics to improve the survival and implantation of beneficial microorganisms in the gastrointestinal tract, have also been studied in the context of T1D. The combination of *Lactobacillus sporogenes* with fructo-oligosaccharides (FOS) for 8 weeks showed a significant decrease in fasting blood glucose, HbA1c and CRP levels, accompanied by a significant increase in mean plasma insulin levels and total antioxidant capacity as compared to the control group (n = 25 in intervention/control group), see Table 4 [153]. However, the combination of short-chain FOS with *L. reuteri*, *L. rhamnosus* and *B. lactis* in T1D patients with albuminuria did not show a protective effect on renal function or inflammatory markers, which could be due to the relatively short treatment duration (12 weeks) and the advanced stage of the disease; in fact, the mean duration of diabetes was 35 years, whereas in the study described above with *L. sporogenes* supplementation, the mean duration was 4 years [154].

### 5.4. Postbiotic-Based Interventions

As already discussed in this review, the metabolites derived from the gut microbiome are crucial mediators of host-microbial interaction and T1D pathophysiology. Rather than focusing on the bacterial species that produce beneficial metabolites, interference with the gut microbiome crosstalk can also be achieved through the use of postbiotics. Postbiotics are defined as “preparations of inanimate microorganisms and/or their components/products that confer a health benefit to the host” [155]. Postbiotic research in human T1D has focused primarily on SCFAs, see Table 5. In a placebo-controlled, double-blind cross-over study in T1D patients, including 30 individuals with long-standing T1D, de Groot et al. investigated the effects of oral supplementation with 4 g/day of sodium butyrate for one month on immune cells, islet autoimmunity, glycemia and beta cell function [156]. Only minimal changes in gut microbiota composition were observed after butyrate supplementation, while no effects were found on stimulated C-peptide levels, HbA1c, daily insulin use, or innate and adaptive immune cell subsets. Nevertheless, butyrate was found to significantly reduce the number of IA2-specific CD8+ T cells, whereas CD8+ T cells specific for other beta cell-derived epitopes were unchanged [156]. A larger study involving 53 T1D patients who received 3.6 g of sodium butyrate or placebo daily for 12 weeks also showed no significant improvement in inflammatory markers, renal parameters, HbA1c, albuminuria and intestinal inflammation after butyrate treatment as compared to placebo [157].

A study of Bell et al. took a different approach by changing the mode of delivery of SCFAs. Following the promising result of HAMSAB in NOD mice [93], a clinical trial was conducted to evaluate the potential therapeutic effect of HAMSAB supplementation for 6 weeks in patients with long-standing T1D [82]. Here, they used high-maize starches modified by acetate and butyrate linkage to deliver a high yield of SCFAs in the colon for 6 weeks, with a 12-week follow-up, in a population of 20 T1D patients with a mean disease duration of 14 years. SCFAs in feces and plasma were significantly increased at both 6 and 12 weeks (following 6 weeks of wash out). This was accompanied by microbiota compositional changes, including significant increases in *Bacteroides uniformis*, unclassified *Parabacteroides* and *P. distasonis* and decreases in *Eubacterium ramulus*, *Eubacterium eligens*, and *Coprococcus comes* after 6 weeks of treatment. Moreover, the HAMSAB diet seems to promote a more tolerogenic environment, as pro-inflammatory cytokines were diminished, while T and B cells exhibited a more regulatory phenotype with increased expression of inhibitory molecules, such as TIGIT and CTLA-4, on T cells, and a decrease in marginal zone-like B cells with high CD86 and major histocompatibility complex-I (MHC-I) expression, suggesting reduced antigen presentation capabilities. Notably, these immunological changes persisted after a 6-week washout period without HAMSAB supplementation. However, HAMSAB supplementation did not improve glycemic control or beta cell function, as fasting glucose and C-peptide were not significantly altered. Nonetheless, subjects with the highest SCFA concentrations had a better glycemic control [82]. It should be noted that this study was conducted in adults with long-standing T1D. Accordingly, a pilot cross-over study of HAMSAB supplementation for 4 weeks in adolescents diagnosed within 2 years showed no change in glycaemia and beta-cell function, assessed by continuous glucose monitoring and C-peptide excursion during the mixed meal test, after HAMSAB or the standard recommended “diabetes diet”. However, the authors did find that HAMSAB supplementation hampers the activation state of MAIT cells, which are innate-like T cells that are involved in the mucosal immune response [158]. A phase Ib double-blinded, placebo-controlled trial enrolling 39 patients (NCT06057454) is underway to evaluate the effects of HAMSAB diet in recently diagnosed T1D patients. The lack of clinical benefits on glycemia and beta cell function in the studies of de Groot et al. and Bell et al. may be attributed to the timing of treatment, as patients with long-standing T1D were selected in this study rather than new-onset T1D patients or at-risk individuals.

### 5.5. Fecal Microbiota Transplantation

Fecal microbiota transplantation (FMT) has served as a valuable experimental tool to establish the transmissibility of disease phenotypes in animal models, such as NOD mice, but has also shown promise as a potential therapeutic strategy to mitigate or prevent T1D development. Beyond isolated case reports involving FMT in T1D, one clinical trial studied FMT in adult patients with recent-onset T1D (<6 weeks) [159]. Participants were randomized into two groups to receive three FMTs, either autologous (n = 10) or allogeneic from a healthy donor. Interestingly, in the autologous FMT group both fasted and stimulated C = peptide levels were significantly preserved 12 months after the first FMT. Subsequent FMTs resulted in changes in the abundance of specific commensal microbes and plasma metabolites, with fecal *Desulfovibrio piger* and plasma metabolites 1-arachidonoyl-GPC and 1-myristoyl-2-arachidonoyl-GPC levels increasing over time in the autologous FMT groups and positively correlating with C-peptide levels. Additionally, FMTs altered the frequency of circulating activated CD4+ CXCR3+ and CD8+ CXCXR3+ T cells, which was inversely associated with C-peptide and *Desulfovibrio piger* rates [159]. Based on promising results, future studies involving transmission of fecal microbiota will move towards an encapsulated form containing frozen or lyophilized fecal microbiota, with the advantage that fecal microbiota capsules (FMC) are non-invasive, less discomfortable and can be more easily used in vulnerable patient populations, such as young children at high risk for T1D or those with new-onset T1D [160]. Currently, our group is investigating the effects of encapsulated autologous FMTs in 10 patients recently diagnosed with T1D (0.5–3.5 years after diagnosis), with the primary endpoint being preservation of residual beta cell function, assessed during mixed meal tests (NCT05323162).

## 6. Conclusions, Discussion and Future Directions

In this review, we have elucidated the intricate role of the gut microbiome in the pathogenesis and potential treatment of T1D, highlighting recent advances in this rapidly evolving field of study.

Future research directions should focus on harnessing the gut microbiome as an adjunctive therapy, emphasizing a personalized medicine approach rather than a one-size-fits-all strategy. However, it is imperative to note that while numerous therapeutic approaches have shown promise in preventing or reversing T1D in NOD mice, the translation of these findings into effective treatments for human T1D has been largely unsuccessful [161]. This discrepancy between preclinical success and clinical outcomes highlights that (I) the NOD mouse model has limitations in fully replicating the complexity of the human immune system and disease, (II) the timing of intervention is crucial, and more investigation is warranted in the early life period, and (III) the heterogeneity of human T1D is higher compared to animal models [161]. The timing of intervention is paramount, necessitating large-scale longitudinal investigations, exemplified by the Environmental Determinants of Islet Autoimmunity (ENDIA) study [162]. The latter aims to identify environmental factors and gene–environment interactions that contribute to the development of islet autoimmunity and T1D by following at-risk infants from early pregnancy into childhood with multiple plasma and fecal samples to avoid picking up natural fluctuations in microbiota compositions [163].

While our focus in this review has been primarily on the bacteriome, it is crucial to acknowledge that the gut microbiome encompasses a broader spectrum of microorganisms, including viruses, fungi, archaea and protists. The dynamics and complexity of these commensal communities, beyond bacteria, warrant further investigation [164]. Beyond the well-studied enteroviruses and other specific candidate viruses, the potential role of unknown viral entities in T1D development remains largely unexplored. Recent evidence has shown significant differences in the gut virome prior to the first signs of T1D, suggesting a possible predictive or causative relationship. In addition, bacteriophages may interact directly with the host, beyond their influence on bacterial populations, adding another layer of complexity to the microbiome–host relationship [164]. Additionally, yeasts and fungi within the gut ecosystem, the mycobiome, represent an emerging area of research in the context of T1D, but their potential impact on disease pathogenesis and progression remains to be fully elucidated [165]. In parallel, efforts could be directed towards the development of novel microbiome-based biomarkers for early detection and prognosis as well as the investigation of potential synergistic effects between microbiome-based therapies and existing immunomodulatory interventions.

In conclusion, the gut microbiome offers valuable insights into the complex pathophysiology of T1D and paves the way for innovative treatment strategies, leading to more effective management and possibly prevention of the disease in the future. However, future research is imperative before gut microbiota-related interventions can be broadly applied in human T1D.

## Figures and Tables

**Table 1:** Summary of the main characteristics of the gut microbiome associated with different stages of type 1 diabetes. The table includes a description of the population (subgroups) studied, the main taxonomic changes in the gut microbiota and the name of the cohort study, first author and reference number.

Population	Main Changes in Gut Microbiota	Reference
Disease stage: high genetic risk, seroconversion to development of T1D		
Young children with high-risk HLA genotype (n = 783), including children who seroconverted (n = 267), T1D diagnosed (n = 101) and controls (n = 415). Finland, Sweden, Germany, USA Age: 3 months until 5 years.	Gut microbiome of control children contained more genes associated with fermentation and biosynthesis of SCFAs. T1D-associated microbial compositions were taxonomically diffuse but functionally more coherent. Children with islet autoimmunity had lower abundance of *Lactobacillus rhamnosus* and *Bifidobacterium dentium* and higher abundance of *Streptococcus* group *mitis/oralis/pneumoniae* species compared to controls. Children developing T1D had lower abundance of *Streptococcus thermophiles* and *Lactococcus lactis* species and higher abundance of *Bifidobacterium pseudocatenulatum*, *Roseburia hominis*, and *Alistipes shahii.*	TEDDY study Vatanen et al., 2018 [61]
Young children with high-risk HLA genotype (n = 903), with children developing islet autoimmunity (n = 632) and T1D (n = 196). Finland, Sweden, Germany, USA. Age: 3 months until 4 years.	Alpha diversity was comparable between cases and matched controls for both islet autoimmunity and T1D. A higher abundance of *Erysipelotrichaceae* was found in children with islet autoimmunity. Five bacterial genera were significantly associated with the onset of T1D, including *Parabacteroides*. Eleven bacterial genera were lower in T1D patients, including *Ruminococcaea*, *Lactococcus*, *Streptococcus*, and *Akkermansia.*	TEDDY study Stewart et al., 2018 [62]
Children at high genetic risk for T1D (n = 33), 11 seroconverted to T1D related autoimmunity, including 4 who later developed T1D, 22 remained healthy (controls). Finland and Estonia. Age: from birth until 3 years.	25% decrease in alpha diversity between seroconversion and T1D progression. Gut microbial gene content is altered prior to the clinical onset of T1D, and changes in both the phylogenetic and metabolic pathway composition of the microbiome were characteristic of a pro-inflammatory environment, with a relative overabundance of *Blautia*, the *Rikenellaceae*, and the genera *Ruminococcus* and *Streptococcus* (not statistically significant) and a relative underabundance of the *Lachnospiraceae* and *Veillonellaceae* (not statistically significant).Additionally, a decrease in the levels of the secondary bile acid lithocholic acid was found.	DIABIMMUNE study Kostic et al., 2015 [36]
Children of ABIS (not at genetic risk) cohort, with future T1D diagnosis (n = 16) and matched healthy controls (n = 32). Sweden. Age: from birth until 20 years, feces samples at age of 1 year.	At 1 year of age distinct microbial signatures were identified, with reduced predicted bacterial SCFA pathways in future T1D children. Genera more abundant in future T1D children were *Firmicutes* (*Enterococcus*, *Gemella* and *Hungatella*), and *Bacteroides* (*Bacteroides* and *Porphyromonas*), whereas in control, *Firmicutes*, such as *Anaerostipes*, *Flavonifractor*, *Ruminococcaceae* UBA1819 and *Eubacterium* were more abundant. *Ruminococcus* was a strong determinant in differentiating future T1D diagnosis and control. *Alistipes* (more abundant in control infants) and *Fusicatenibacter* (mixed abundance patterns when comparing case and control infants) were the strongest factors for differentiating future T1D.	ABIS study Bélteky et al., 2023 [64]
Children at high genetic risk for T1D (n = 76), 29 seroconverted to T1D related autoimmunity, including 22 who later developed T1D, 47 remained healthy (controls). Finland. Age: 4 months until 2.2 years.	Abundance of *Bacteroides dorei* was significantly higher preceding the appearance of islet autoimmunity.	DIPP study Davis-Richardson et al., 2014 [65]
Individuals with recent-onset T1D (n = 33), islet autoantibody-positivity (n = 17), low-risk autoantibody negativity (n = 29) and healthy subjects (n = 22). USA. Age: 2–45 years.	Microbial-derived proteins were able to discriminate new-onset T1D and islet autoantibody positivity from low-risk individuals.T1D patients had increased intestinal inflammation and decreased barrier function along with a depletion of microbial taxa associated with host proteins involved in maintaining mucosal barrier function and microvilli adhesion, which was associated with a decrease in *Alistipes*. Exocrine pancreatic dysfunction was found in both T1D patients and high-risk individuals prior to disease onset.	Gavin et al., 2018 [69]
Disease stage: seroconversion to islet autoimmunity (at risk of developing T1D)		
Children at high genetic risk for T1D (n = 74), of whom children developed single islet autoantibody but did not progress to T1D during follow-up (n = 23), developed multiple islet autoantibodies with high risk for progression to T1D (n = 13) and controls (n = 38). Finland, Russia and Estonia. Age: from birth until 3 years.	At an early age, systemic bile acids and microbial secondary bile acid pathways were altered in the multiple islet autoantibody group, compared to single autoantibody development and controls. Within this group, lower levels of *Clostridium* and *Eggerthella lenta* and increased levels of *Runinococcus* strains were found at 18 months and 24 months of age.	DIABIMMUNE study Lamichhane et al., 2022 [63]
Children with at least two diabetes-associated autoantibodies (n = 18) compared to age/sex/early feeding history and HLA risk genotype matched antibody-negative children (n = 18). Finland. Age: 3.9—14.2 years.	Higher relative abundance of *Bacteroidetes* and various *Firmicutes* (*Clostridium perfringens*) in children with autoantibodies, lower levels of lactate- and butyrate-producing bacteria and *Bifidobacterium adolescentis* and *Bifidobacterium pseudocatenulatum* (<12% combined).	FINDIA/TRIGR trials De Goffeau et al., 2013 [67]
Young children with islet autoimmunity (n = 22) and healthy controls (n = 22). Germany. Age: 0.2 until 3.2 years.	Gut microbiome profiles characterized by *Bacteroides* dominance and altered mucin-degrading bacterial communities were associated with the development of islet autoantibodies and reduced genetic capacity for butyrate production.	Endesfelder et al., 2016 [72]
Children with a first-degree relative with T1D (n = 40), with children developing persistent islet autoantibodies (n = 20) and controls matched for age, sex and HLA (n = 20) Australia. Age: 0–8 years	The onset of islet autoimmunity was accompanied by a decrease in the abundance of members of the *Ruminococcaceae* family. In addition, islet autoimmunity is associated with a functional remodeling of the gut microbiota with a switch in the function of *Faecalibacterium* and Bacteroidetes.	Gavin et al., 2022 [75]
Disease stage: new-onset T1D		
Children with new-onset T1D (n = 64) and healthy controls (n = 77) matched for age, sex, delivery and feeding mode. China. Age: 4–10 years.	Decreased butyrate-producing species such as *Faecalibacterium prausnitzii*, *Eubacterium rectale*, and *Roseburia intestinalis* and enrichment of opportunistic pathogens such as *Escherichia coli*, unclassified *Enterobacteriaceae*, and *Klebsiella pneumoniae* in T1D patients compared to controls. In addition, a decrease in bile acid metabolism and an increase in LPS biosynthesis were found in T1D patients compared to controls. Fecal microbiota transplantation (FMT) from T1D patients to antibiotic-treated mice induced impaired glucose homeostasis.	Yuan et al., 2022 [35]
Newly diagnosed T1D (n = 98) and autoantibody-positive unaffected family members (n = 194). Europa. Age: 12.3 ± 8.6 years.	A longitudinal increase in 21 bacterial species was found in newly diagnosed T1D patients. A significant negative association was found between the relative abundance of butyrate-producing *Faecalibacterium prausnitzii* and HbA1c levels at diagnosis. The rate of the subsequent disease progression was associated with the abundance of several bacterial species and individuals with a rapid decline in C-peptide levels, reflecting a decline in endogenous insulin production, had the lowest gut microbiome diversity.	INNODIA study Vatanen et al., 2024 [66]
Children and adolescents shortly after T1D onset (n = 73) and healthy controls matched for age and geographics (n = 104). Azerbaijan, Jordan, Nigeria, Sudan. Age: 7.8–14 years	Significant positive association with T1D for the genus *Escherichia* (class *Gammaproteobacteria*, phylum Proteobacteria), while butyrate-producing *Eubacterium* and *Roseburia* (class *Clostridia*, phylum Firmicutes) were inversely associated with T1D.	Cinek et al., 2018 [73]
Children with T1D (n = 16) and healthy controls (n = 16) Spain. Age: 6–8 years.	Significantly decreased Firmicutes:Bacteroidetes ratio in T1D patients, with Bacteroidetes significantly increased and Actinobacteria and Firmicutes significantly decreased compared to control. At the genus level, a significant increase in the number of *Clostridium*, *Bacteroides* and *Veillonella* and a significant decrease in the number of *Lactobacillus*, *Bifidobacterium*, *Blautia coccoides*/*Eubacterium rectale* group and *Prevotella*, important bacteria for butyrate production, were found in T1D patients. The number of *Bifidobacterium*, *Lactobacillus*, *Clostridium* and the ratio of Firmicutes to Bacteroidetes correlated significantly with the plasma glucose levels.	Murri et al., 2013 [70]
Teenagers and young adults with T1D (n = 20) and healthy controls (n = 28). Brazil. Age: 23.1 ± 8.6 years	Lower beta diversity in T1D patients compared to controls. *Bacteroides* and *Bilophila* abundances were higher in T1D patients, whereas *Streptococcus* and Ruminococcaceae were lower. *Faecalibacterium* abundance correlated inversely with HbA1c levels (poor glycemic control).	Higuchi et al., 2018 [74]
Disease stage: long-standing T1D		
Adults with T1D (n = 53) and healthy controls matched for age, sex and BMI (n = 50). The Netherlands. Age: 18–65 years.	Gut microbiota had lower butyrate-producing species and less butyryl-CoA transferase genes in adults with T1D, and plasma levels of acetate and propionate were lower. *Christensenella* and *Subdoligranulum* strains correlated with glycemic control, inflammatory parameters and SCFAs.	De Groot et al., 2017 [68]
T1D patients with average disease duration of 28 ± 15 years (n = 238) and healthy controls matched for age, sex and BMI (n = 2937). The Netherlands. Age: of 52 ± 16 years.	No differences in alpha diversity of microbial taxa, but significant increase in alpha diversity of microbial biochemical pathways in T1D patients compared to controls. Significant differences in microbial species, including depletion of *Alistipes putredinis*, *Prevotella copri* and *Bifidobacterium longum* and enrichment of *Ruminococcaceae*, *Clostridiaceae*, *Clostridiales* and genus *Oscillibacter.* HbA1c and disease duration as well as micro- and macrovascular complications could explain a significant part of the variation in the gut microbiome.	Van Heck et al., 2022 [71]

**Table 2:** Clinical intervention using prebiotics in T1D treatment.

Intervention	Population	Main Changes	Reference
Oligofructose-enriched inulin vs. placebo for 12 weeks. Randomized placebo-controlled trial.	Children with T1D (n = 17 prebiotic, n = 21 control). Age 12.5 ± 2.8 years.	Significant increase in relative abundance of *Bifidobacterium*, decrease in *Streptococcus*, *Roseburia inulinivorans*, *Terrisprobacter* and *Faecalitalea*. C-peptide was significantly higher after prebiotics, along with a significant improvement in intestinal permeability as measured by a lactulose/mannitol test. There was no significant change in HbA1c or inflammatory markers.	Ho et al., 2019 [137]

**Table 4:** Clinical intervention studies implementing synbiotics (combination of probiotics and prebiotics) in T1D.

Intervention	Population	Main Changes	Reference
*Lactobacillus sporogenes* GBI-30 (probiotic), maltodextrin and fructo-oligosaccharides (prebiotic) vs. placebo for 8 weeks. Randomized, placebo-controlled, double-blind trial.	Adults with T1D (symbiotic n = 25, placebo n = 25).	Significant reduction in HbA1c and CRP in the intervention group. Fasting blood glucose was significantly lower in the probiotic group compared to control. Significant increase in mean plasma insulin levels and total antioxidant capacity.	Zare Javid et al., 2020 [153]
Short chain fructo-oligosaccharides and *L. reuteri*, *L. rhamnosus* and *B. lactis* and placebo, both for 12 weeks. Randomized, placebo-controlled, crossover study.	Adults with T1D and albuminuria (n = 35). Age 58 ± 11 years.	No significant effect on albuminuria, endothelial function, kidney function, blood pressure and markers of low-grade inflammation.	Stougaard et al., 2024 [154]

**Table 5:** Clinical intervention studies, in which various forms of short-chain fatty acids were administered to T1D individuals.

Intervention	Population	Main Changes	Reference
Oral sodium butyrate (4 g) and placebo, both for 4 weeks. Randomized, double-blind, placebo-controlled, crossover trial.	Adults with T1D, with mean duration 8 years (n = 30). Age 32.5 (22–61) years	Significant reduction in fecal butyrate and propionate levels. No significant change in alpha diversity. Higher abundance of several *Lachnospiraceae* species, while *Lachnospiraceae Blautia*, *Lachnospiraceae Marvinbryantia*, *Lachnospiraceae* NK4A136 group and *Faecalibacterium prausnitzii* were less abundant. No change in glucose metabolism or beta cell function. No significant changes in adaptive or innate immunity.	De Groot et al., 2022 [156]
Oral sodium butyrate (3.6 g) vs. placebo for 12 weeks. Randomized, double-blind, placebo-controlled trial.	Adults with T1D (postbiotic n = 28; placebo n = 25). Age 54 ± 13 years.	No significant changes in fecal calprotectin, SCFAs, inflammatory markers, kidney parameters, HbA1c or gastrointestinal symptoms.	Tougaard et al., 2022 [157]
HAMSAB supplementation for 6 weeks, 40 g/day. Single-arm pilot study.	Adults with long-standing T1D with a mean disease duration of 14 years (n = 20). Age 18–45 years.	Increase in SCFAs in feces and plasma. Shift in gut microbiome composition and function with significant decrease in alpha diversity, with changes in *Parabacteroides distasonis*, *Bacteroides ovatus*, *Bifidobacterium adolescentis*, and *Dialister invisus.* No significant change in fasting glucose or C-peptide levels. Subjects with the highest SCFA concentration had the best glycemic control. T cells exhibited a more regulatory phenotype with a decrease in several pro-inflammatory cytokines.	Bell et al., 2022 [82]
HAMSAB supplementation for 4 weeks vs. control diet. Cross-over design, Phase Ib study.	Recently diagnosed (<2 years of diagnosis) youths with T1D (n = 7). Age 15.0 ± 1.2 years.	Significant increase in fecal butyrate levels. Increase in the relative abundance of *Bifidobacterium* and *Faecalibacterium*. Reduced activation of mucosal-associated invariant T cells. Significant reduction in the area under the curve glucose levels in the mixed meal tolerance test, but not for C-peptide.	Ismail et al., 2025 [158]

## Data Availability

Not applicable.
